# Acceptance and use of a clinical decision support system in musculoskeletal pain disorders – the SupportPrim project

**DOI:** 10.1186/s12911-023-02399-7

**Published:** 2023-12-19

**Authors:** Fredrik Granviken, Ingebrigt Meisingset, Ottar Vasseljen, Kerstin Bach, Anita Formo Bones, Nina Elisabeth Klevanger

**Affiliations:** 1 Department of Public Health and Nursing, Norwegian University of Science and Technology (NTNU), Postboks 8905, Trondheim, 7491 Norway; 2 Clinic of Rehabilitation, St. Olavs Hospital, Trondheim, Norway; 3 Unit for Physiotherapy Services, Trondheim Municipality, Trondheim, Norway; 4 Department of Computer Science, Norwegian University of Science and Technology (NTNU), Trondheim, Norway

**Keywords:** CDSS, Clinical Decision Support System, Musculoskeletal pain disorders, Acceptance, Usability, Physiotherapy, Primary Care, Interview, Qualitative

## Abstract

**Background:**

We have developed a clinical decision support system (CDSS) based on methods from artificial intelligence to support physiotherapists and patients in the decision-making process of managing musculoskeletal (MSK) pain disorders in primary care. The CDSS finds the most similar successful patients from the past to give treatment recommendations for a new patient. Using previous similar patients with successful outcomes to advise treatment moves management of MSK pain patients from one-size fits all recommendations to more individually tailored treatment. This study aimed to summarise the development and explore the acceptance and use of the CDSS for MSK pain patients.

**Methods:**

This qualitative study was carried out in the Norwegian physiotherapy primary healthcare sector between October and November 2020, ahead of a randomised controlled trial. We included four physiotherapists and three of their patients, in total 12 patients, with musculoskeletal pain in the neck, shoulder, back, hip, knee or complex pain. We conducted semi-structured telephone interviews with all participants. The interviews were analysed using the Framework Method.

**Results:**

Overall, both the physiotherapists and patients found the system acceptable and usable. Important findings from the analysis of the interviews were that the CDSS was valued as a preparatory and exploratory tool, facilitating the therapeutic relationship. However, the physiotherapists used the system mainly to support their previous and current practice rather than involving patients to a greater extent in decisions and learning from previous successful patients.

**Conclusions:**

The CDSS was acceptable and usable to both the patients and physiotherapists. However, the system appeared not to considerably influence the physiotherapists' clinical reasoning and choice of treatment based on information from most similar successful patients. This could be due to a smaller than optimal number of previous patients in the CDSS or insufficient clinical implementation. Extensive training of physiotherapists should not be underestimated to build understanding and trust in CDSSs.

**Supplementary Information:**

The online version contains supplementary material available at 10.1186/s12911-023-02399-7.

## Background

Musculoskeletal (MSK) pain disorders are the leading cause of disability worldwide and a major societal burden [1]. Most interventions for musculoskeletal pain provide small to moderate short-term effects [2, 3]. Treatment guidelines are based on clinical trials with carefully selected patients and mean group effects, with little consideration for the variability in patients’ symptoms, prognostic factors, and comorbidities, challenging transferability to clinical settings [4]. Focus on person-centred care based on the patient’s preferences and shared decision-making is recommended [5]. Further, more emphasis on prognostic factors has been recommended for treatment decisions and outcome improvement [6]. However, making decisions on the best treatment approach for an individual patient with many and various factors influencing the patient’s trajectory and outcome remains challenging for clinicians.

Clinical decision support is an overarching term including various tools and interventions, ranging from simple non-computerised tools for information management to advanced computerised systems providing patient-specific recommendations [7]. Clinical decision support systems (CDSS) aid clinical decision-making by matching patient characteristics against a knowledge base for patient-specific assessment or treatment recommendations [7–9]. Systematic reviews of CDSSs have shown inconclusive outcomes in healthcare [10–21]. Improvements in practitioner performance [12, 17, 20] and patient outcomes [10, 18] have been reported, whereas others have reported conflicting or no evidence for improvements in medication prescription, medication intake adherence, rate of imaging referrals and practice for ordering of laboratory tests [19].

The effect of CDSSs in daily clinical practice depends on the implementation and usability, including integration into clinical workflow, electronic templates and providing recommendations at the point of care [13]. Further, many systems have challenges realising their full potential with low user acceptance, partly because they lack end-user feedback [15, 22]. Khairat et al. [23] identified 11 studies using qualitative methods to evaluate user acceptance of CDSSs. Facilitators included ease of use, time-saving, and perceived usefulness, while barriers were workflow interference, questionable validity, disturbances, and lack of efficiency. Perceived usefulness has been shown to be the most important factor for implementing complex decision support systems in personalised management of neck and low back pain [24]. Among MSK disorders, one scoping review of clinical decision support tools to help decision-makers select interventions has been published [25]. It concluded that the tools, models, or systems should be subjected to further validation before they are ready for widespread use in clinical practice to select interventions for patients with MSK disorders.

We developed a CDSS based on methods from artificial intelligence (AI) to support physiotherapists and patients in the decision-making process of managing musculoskeletal pain disorders in primary care physiotherapy. The CDSS uses case-based reasoning (CBR), an AI method, to find the most similar successful patients from the past to give treatment recommendations for a new patient [26]. Using previous patients with successful outcomes to advise treatment for new patients moves management of MSK pain patients from one-size fits all recommendations to more individually tailored treatment. This study aimed to summarise the development and explore the acceptance and use of the CDSS for MSK pain patients.

## Methods

### Design, participants, and settings

This qualitative study was performed in advance of a randomised controlled trial evaluating the effectiveness of the CDSS in physiotherapy care. We here describe the results from qualitative interviews exploring the acceptance and use of the CDSS among physiotherapists and their patients. The reporting follows the consolidated criteria for reporting qualitative research (CORE-Q) [27].

The study was conducted in the physiotherapy primary healthcare sector of Norway in October and November 2020. Four physiotherapists were recruited. The physiotherapists were recruited from a pool of physiotherapists being involved in a former longitudinal observational study following patients through their physiotherapy treatment periods in primary health care in Norway [28]. As such, they had no particular interest in the current study. The physiotherapists were purposively selected based on the criteria of covering different physiotherapy specialties, sex, age and experience as a physiotherapist. They worked in both small and large clinics located both in and outside the city. The physiotherapists consecutively recruited a convenience sample of three patients each (a total of 12 patients) who were treated by the recruiting physiotherapist. To educate physiotherapists in the CDSS, we made educational materials consisting of step-by-step written information and videos on how to use the various screens in the CDSS. In addition, we had a one-hour one-to-one digital meeting with all physiotherapists to go through the CDSS together, address specific parts and answer any additional questions. When the patients booked an appointment with the physiotherapist, they were asked if they could be contacted by a researcher for participation in the testing of the CDSS prototype. They were contacted by phone and email with information about the project. Patients who met the inclusion criteria were consecutively recruited by the physiotherapists, signed the consent form online, and completed the baseline questionnaire before the consultation.

Inclusion criteria were patients aged 18 years and above presenting to a primary care physiotherapist with musculoskeletal pain disorder as primary contact reason in any of these areas; shoulder, neck, upper/low back, hip, knee, or multisite/complex pain. Exclusion criteria were reduced cognitive function or skills in Norwegian (impeding reading, speaking, or comprehension of the Norwegian language), pregnancy or pregnancy-related disorders, ongoing cancer, patients scheduled for surgery, surgery or fracture within the last six months, or neurological diagnosis (e.g., multiple sclerosis, stroke, ALS, Parkinson, dementia).

### Theoretical framework

Inspired by the Technology Acceptance Model [29, 30], we explored the acceptance of the CDSS among physiotherapists and patients. User acceptance has two fundamental determinants: perceived usefulness and ease of use (usability). Perceived usefulness concerns whether physiotherapists and patients perceive the CDSS to be useful for what they want to do. Since physiotherspists’ and patients’ goals might not always align, usefulness could be perceived differently. Perceived ease of use addresses the degree of ease associated with using the CDSS for physiotherapists and patients.

### Development

Development of the CDSS followed the guidance for reporting intervention development studies in health research (GUIDED) [31] and principles of agile software development, where developmental phases are revisited and iteratively improved by user feedback [32, 33]. The development of the clinical dashboard for the CDSS consisted of multiple steps. First, we conducted a requirements analysis among stakeholders to define their needs and how to solve various problems. We interviewed user representatives, including two patients and three physiotherapists from different specialities, to map their views regarding the first consultation, important aspects to consider in the first meeting and suggestions for improving consultations. Further, we made functional specifications for the software developers by creating seven fictive users (personas), three patients, and four physiotherapists, representing a broad range of different user characteristics. To ensure a common understanding of the requirements of the CDSS between software developers, physiotherapists, and the researchers, we recorded a video simulating a typical first consultation at a physiotherapy clinic and made several mock-up suggestions for the clinical dashboard of the CDSS. The development of the CBR system and the system’s ability to identify similar patients has been thoroughly described in a former study [26]. The function specifications were defined in system architecture, user interface, and server (back-end) components in the design phase. The user interface was developed through an iterative process with suggestions, feedback, and clarifications between the researchers and developers. Testing was then carried out on user representatives who provided feedback on elements that worked well and suggested changes, which we tried to incorporate. This phase also contained testing and debugging of the entire CDSS.

### The clinical decision support system

**Fig. 1:**
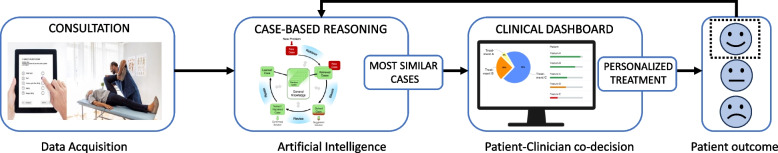
Overview of the flow through the clinical decision support system. Self-reported data and findings from the clinical examination are used to find similar patients with successful outcomes using case-based reasoning (CBR). A clinical dashboard is used for making shared decisions. The self-reported data from the patient and description of the treatment and patient outcome are retained for future problem-solving

AI, in particular CBR [34], was used to build the CDSS (Fig. 1). The CBR methodology’s core underpinning is matching new patients to previous, similar patients with a suitable treatment plan. The output from the CBR is displayed in a clinical dashboard for shared decision-making and to support the optimal management of new patients with common musculoskeletal disorders. The CDSS is based on 105 patients, from which the system identifies the three most similar previous patients with positive outcomes. A positive outcome is defined by a composite score of biopsychosocial domains, including different combinations of pain intensity, function, workability, global perceived effect, and The Musculoskeletal Health Questionnaire (MSK-HQ) [35].

**Fig. 2:**
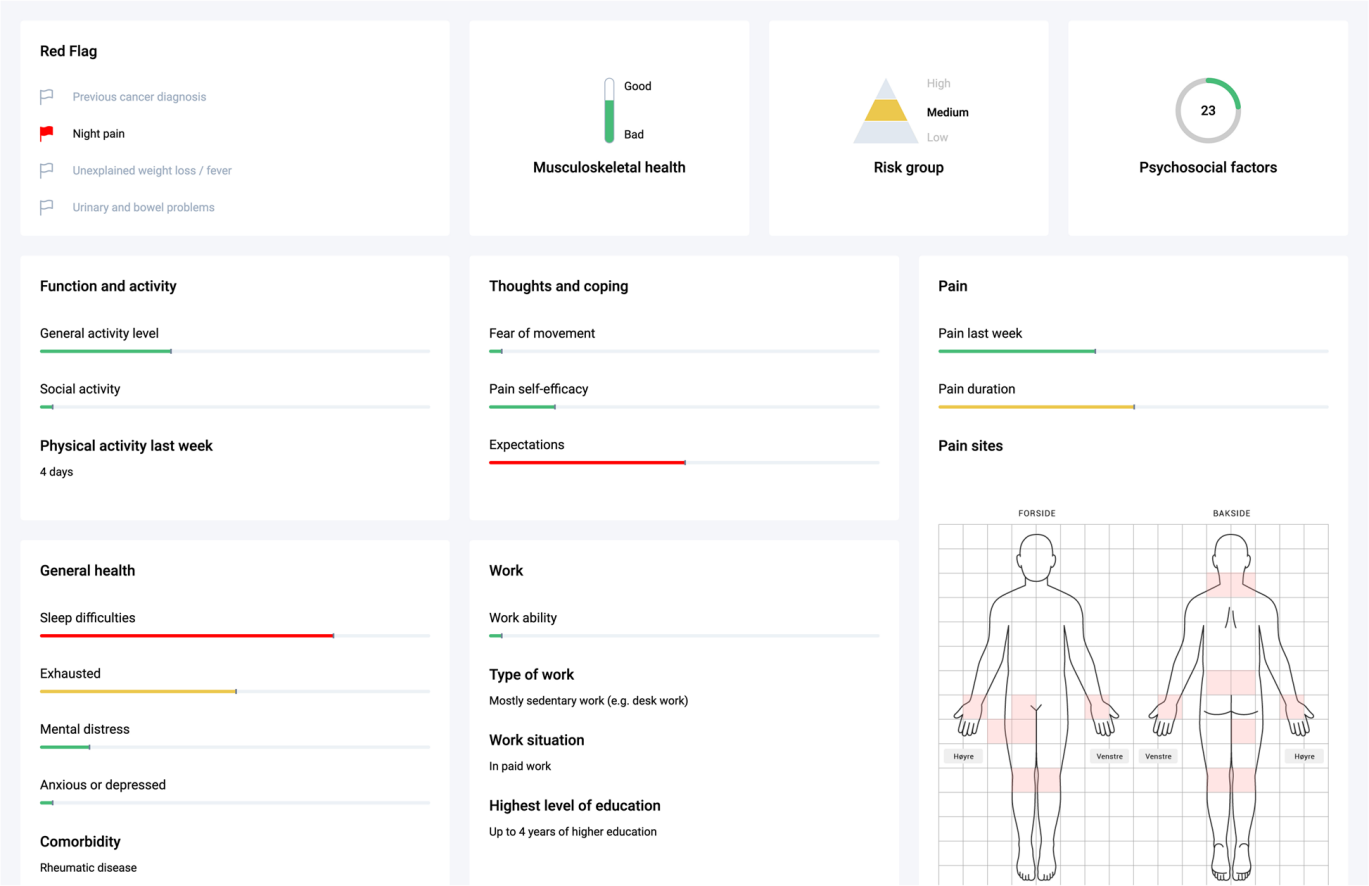
The patient profile as it is displayed in the clinician dashboard

**Fig. 3:**
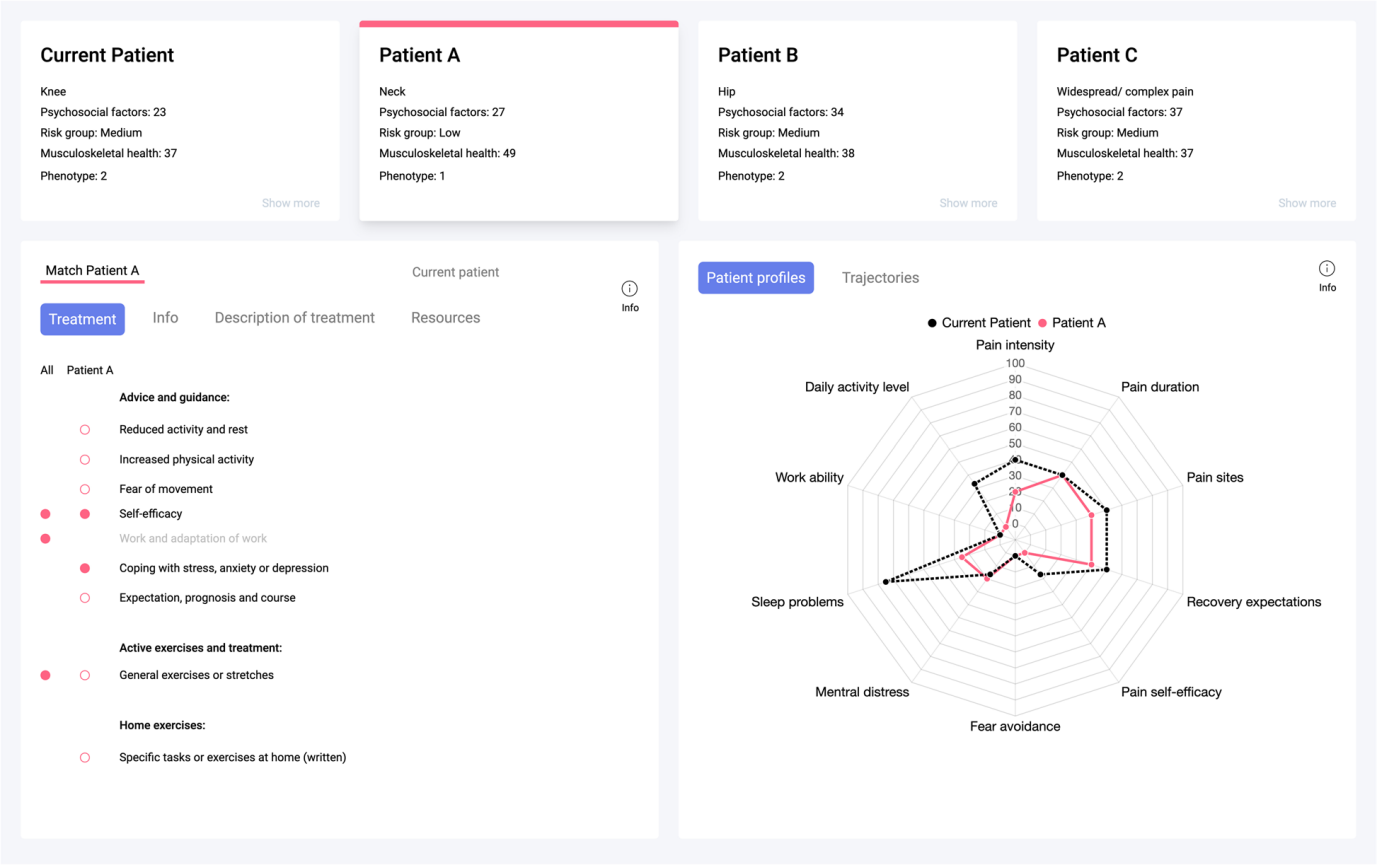
The treatment decision screen

At the first consultation, the physiotherapist controls the clinician dashboard and views this together with the patient. The clinical dashboard (Fig. 1) consists of two main components; the patient profile (Fig. 2) and the treatment decision screen (Fig. 3). The patient profile graphically displays the patient's symptom severity on important prognostic factors self-reported by the patient before the first appointment, including the overall scoring for musculoskeletal health (The Musculoskeletal Health Questionnaire [35]), the patient’s risk group affiliation (The STarT MSK Tool [36]), and overall scoring on psychosocial factors (The Short Form Örebro Musculoskeletal Pain Screening Questionnaire [37]). Symptom severity is displayed with a traffic light system where red refers to high, yellow to medium, and green to no or minor symptom severity. In addition, the patient’s self-reported answers on important red flag questions are highlighted if positive (Fig. 2).

During the first consultation, the physiotherapist examines the patient and registers the findings from the examination in the clinical dashboard (not shown). The treatment decision screen displays the current patient and the three most similar patients with successful outcomes (Fig. 3). Together the physiotherapist and patient decide which of the three patients to learn from. Matching patients can be compared through overall scores in musculoskeletal health, risk group affiliation, overall scoring on psychological factors and phenotypes [38] or by a radar plot with important prognostic factors of the current patient and any of the three most similar patients (Fig. 3). Three months of clinical trajectories of function, workability, pain intensity, pain self-efficacy, and global improvement for similar patients can also be displayed. Further, the physiotherapist and patient agree on a treatment plan based on the treatment given to the selected patient (best match). Treatment options from the CDSS include both free text of the treatment, and information on how much the different domains was emphasised. The domains include treatment advice and education (e.g., physical activity, sleep, work, social participation and coping with stress, anxiety, depression etc.), active treatment (e.g., strength and endurance training), passive treatment (e.g., mobilisation and manipulation), cooperation with others (e.g., GP’s, medical specialists, psychologists) and information on how the treatment has been delivered (group setting, individual consultations, or home exercises). In addition, evidence-based internet resources with advice pertaining to the patient’s condition can be accessed for recommendations. For example, if the patient has trouble sleeping, advice on managing sleep problems is displayed, and links to patient information on the internet can be accessed and printed.

### Interviews

Semi-structured individual interviews were conducted by phone, audiotaped, and transcribed verbatim to assess the patients' and physiotherapists' experience using the CDSS. The interview guide for physiotherapists predominantly consisted of questions regarding the perceived usefulness and usability of the CDSS, including the patient profile, treatment screen and system integration in the clinical workflow. The interview guide for the patients consisted of questions regarding their experiences with filling out the questionnaire at home, the content of the various components, involvement in decisions and general impressions. We also asked for suggestions for improvement of the CDSS. The interview guides can be found in the Appendix Additional file 1. Three researchers (FG, AB, NEK) conducted the interviews; FG and NEK alternated in leading while the other interviewer(s) made notes and provided follow-up questions. We found it helpful to email the patients screenshots of the dashboard in advance to use as references during the interview. The physiotherapists were logged into the CDSS during the interviews for the same purpose. As the interviews commenced and we identified new themes, some questions were added to the interview guides.

### Data analysis

Interviews were analysed thematically using the Framework Method which is often used in health research for thematically analysis of interview data [39]. The procedure for analysis included 1) transcription and familiarisation with data by reading and re-reading transcript interviews, 2) coding with open coding and agreement on codes and themes for an initial analytic framework, 3) developing a working analytic framework with refinement and agreement on codes and themes, 4) applying the analytic framework in all interviews, 5) charting data into framework matrix in Microsoft Excel, and 6) interpreting the data. Researchers (FG, NEK, AB) worked independently by coding the two first interviews before comparing and reaching an agreement for the initial analytic framework. FG coded the remaining interviews and adjusted the working analytic framework when needed. FG, NEK, and AB further discussed and agreed on the themes. All authors contributed to interpreting the data, and FG drafted the results.

### Ethical considerations

The Regional Committee for Medical and Health Research Ethics in Central Norway approved the study (49308/2020). The physiotherapists verbally confirmed participation, and all patients provided written informed consent. All methods were carried out in accordance with relevant guidelines and regulations.

## Results

Twelve patients participated in the study, a majority were women (75%), and most were married or living with others (67%) (Table 1). There were two females and two males among the four physiotherapists, and they worked in three different clinics. They were aged 31, 60, 66, and 67 years old. Two were general physiotherapists, one was a specialist in manual therapy, and one was a specialist in psychomotor physiotherapy [40]. Three were considered very experienced, working as physiotherapists for more than 20 years, and one was less experienced, working less than five years. The physiotherapists had some experience using questionnaires in previous research projects.

**Table 1:** Patient characteristics from the baseline questionnaire

Characteristics	*n* = 12
Age (years), n	
30–39	3
40–49	3
50–59	1
60–69	4
Above 70	1
Sex (female), n (%)	9 (75)
Married or living with others, n (%)	8 (67)
Education, n	
Primary school or less	1
High school	3
Up to 4 years of higher education	5
More than 4 years of higher education	3
Employment, n	
Sick leave	3
Disability pension or work assessment allowance	2
Paid employment	5
Retired	2
Health literacy- difficulty understanding health information, n	
Never	3
Rarely	8
Occasionally	1
Often	0
The main problem, n	
neck	1
shoulder	2
back	3
hip	3
knee	0
complex	3
Pain duration more than one year, n (%)	8 (67)
Pain intensity last week (0–10)^a^, median (range)	5.5 (2–7)
MSK-HQ (0–56)^b^, median (range)	37.5 (24–46)
ÖMSPQ (0–100)^c^, median (range)	47.0 (32–71)

^a^Örebro item 2: “How would you rate the pain you have had during the past week?” [37]

^b^The Musculoskeletal Health Questionnaire (MSK-HQ) [35]

^c^The Short Form Örebro Musculoskeletal Pain Screening Questionnaire (ÖMSPQ) [37]

### Results from the interview analysis

From the analysis of the interviews, we identified five themes: (1) Overall impression of usability and acceptability (facilitators and barriers), (2) A tool for exploring and preparing, (3) Building a therapeutic relationship, (4) Patient involvement, and (5) Recovery expectations (Table 2).

**Table 2:** List of themes and codes

Themes	Codes
**1. Overall impression of usability and acceptability**	
Patient profile	*Understandable, self-explanatory, informative, acceptable amount of information, good design (figures, symbols, text, colour), easy to use, well-structured, reflects how the patient history is currently taken (anamnesis) and the current workflow, possible to integrate, enough/not enough time, quick picture of the patient and focus, relevant for prognosis*
Matching and treatment	*Means to discuss patient preferences, valuable with multiple factors, interface: messy with excessive, irrelevant, confusing information, treatment suggestions: too extensive/too sparse, unsuitable, requiring explanation if to include patient (or not used)*
**2. A tool for exploring and preparing**	
Patients	*Data collection beneficial: less need for explanation, saves time, relief: do not forget, enables targeted questions, otherwise hard to open up, increased reflexivity: more aware, empowering, thinking differently about health, effects on sleep, work and social life, more/less severe than expected leading to questioning own coping skills/relief, physiotherapists seem prepared, enthusiastic, and reach heart of the matter faster*
Physiotherapists	*Facilitates anamnesis and comprehensive thinking, maps all relevant aspects of health, careful: can shape therapists’ behaviour, should not rely on the system*
**3. Building a therapeutic relationship**	
Physiotherapists	*Good starting point for conversations, useful in building therapeutic relations, structures anamnesis, dialogue and focus, careful: prejudice if system shows high and multifaceted symptoms, cautious, not become deterministic*
Patients	*First impression: felt seen and heard, not another in line, involved, physiotherapist explained in an understandable language, CDSS: makes it easier to explain, aligns understandings of the problem and treatment, already aware: visualisation is not scary/depressing*
**4. Patient involvement**	
Patient profile and goals	*Patient participation, elaboration, dialogues and discussions, joint goal- and activity setting, increases awareness and feeling of purpose*
Matching and treatment	*Varying involvement dependent on therapist and patient, high: actively participating in selection, discussing alternatives, deciding, low: not seen the screen/similar patients, physiotherapist decided treatment, patients: good feeling to participate, opportunity to omit, add, or question treatment and have it explained, included more than expected, could have used more time, surprised by activity level, rely on physiotherapists expertise, physiotherapists: support or substantiate treatment already decided/own practice, tool for maintaining biopsychosocial focus, reassess best practice, uncertain addressing some issues (mental health, sleep), open vs inappropriate to adapt some treatments, careful: can spread uncertainty when suggestions differ*
**5 Recovery expectations**	
	*Reassuring: someone similar/worse off has improved, not reassuring: similar matches are much older, patient trajectory graphs: motivating, timespan for and variations in improvement, can lead to positive beliefs, recovery expectations, careful: can create unrealistic expectations*

#### 1) Overall impression of usability and acceptability (facilitators and barriers)

Both patients and physiotherapists reported that the language used in the CDSS was understandable and informative. Overall, physiotherapists described the CDSS as well-structured, simple to use, and possible to integrate into a first consultation with a patient. Some reported that the structuring of the CDSS reflected their current workflow in consultations. The CDSS was used in one to three consultations, in most cases two, at the start of the treatment period, for each patient. In the first consultation, the focus was on the patient profile and the examination as a part of the anamnesis. The second focused on finding the most similar patients and deciding the treatment. Physiotherapists reported that the time used on the CDSS was acceptable, with 10- 30 min on the patient profile, resembling the time usually spent on anamnesis, and 10- 15 min on the treatment decision screen. However, patients were divided on whether they had spent enough time on the different parts of the CDSS.

Both physiotherapists and patients were particularly positive about the usability of the patient profile. The combination of figures, text, symbols, and colours was perceived as nicely presented, easy to understand, self-explanatory and relevant for knowledge about the prognosis: *“The use of colour was brilliant, both for me and not least for the physio. She understood what the problem was and I thought it matched very well with what I had answered” [Patient 3].* The patient profile gave a quick and clear picture of the patient's state of health. In addition, the amount of information presented in the profile was acceptable for the physiotherapists and most patients.

The user acceptance of finding similar patients for treatment suggestions was, on the other hand, more divided. When viewing the three best matches, some physiotherapists underlined the value of having multiple factors to aid in selecting the most similar patient and discussing with the patient which to pursue. However, some user interface elements were described as messy, containing excessive, irrelevant, or confusing information that patients found challenging to understand, thus requiring thorough explanation: *“The patient matching was more difficult to understand. What is meant by towards the middle and what is meant by towards the side of the figure [radar plot], although I tried to explain what it was, it was not easy to understand” [Physiotherapist 1].* The functionality of finding appropriate treatment based on treatment descriptions from previous patients also received variable feedback. Treatment descriptions were described as sometimes too extensive or too sparse to learn from, unsuitable for the patient’s problem, or requiring explanation from the physiotherapist to enable the patient to participate in finding treatment. If not considered relevant for the patient at hand, the physiotherapists refrained from showing the descriptions to the patients. *"Challenging with the figures and the scores. I didn't really see the numbers, so I didn't know why they were there, I didn't understand where zero was" [Patient 10].*

#### 2) A tool for exploring and preparing

The patients reported that gathering information before the first consultation through the baseline questionnaires was beneficial. They appreciated that the physiotherapist had background knowledge of their situation before arriving at the clinic since they then did not have to explain everything in the consultation, thereby saving time. Some felt a sense of relief having filled in the questionnaires, either due to a fear of forgetting something potentially important or by having to address difficult topics when meeting someone new: *"Sometimes you can be afraid to open up about difficult things in consultations like this, and then it can be a support that the physiotherapist knows the background and can ask targeted questions” [Patient 11].*

After filling in the questionnaire, patients described that they had become more aware of topics to focus on with the physiotherapist and thought differently about their health. Overall, the patients were empowered and felt positive about increasing their reflectivity on how the situation affected their sleep, work, and social activity. Some described a sense of relief by not suffering from anything serious: *“I felt pretty healthy when I saw what people could be bothered with, so then I felt pretty good” [Patient 07].* On the other hand, recognising that the situation was more severe than suspected, one patient became afraid of the scope and progression of her pain, questioning her coping skills: *“I am so used to living with pain that I think I'm coping quite well. That's why I got confused when I saw that I wasn't coping as well as I thought I did” [Patient 1].*

The patients perceived the therapists as enthusiastic to the CDSS and well prepared, which allowed them to go to the heart of the matter faster due to the information they had. All physiotherapists said they appreciated having information available before the first consultation to get to know the patient and prepare for the consultation: *“You can say that it makes the anamnesis easier, and you also feel that you are a little familiar with the patient in advance” [Physiotherapist 1].* They believed the CDSS would help to guide them to think more comprehensively about their patients by mapping all relevant aspects of their health situation, facilitating the anamnesis. However, one physiotherapist was sceptical of how it could shape physiotherapists´ questions and further investigation and warned against relying too much on the information.

#### 3) Building a therapeutic relationship

Using the patient profile together was described by both patients and physiotherapists as a good starting point for conversations and getting to know each other: *"It is a good starting point. Sitting together and looking at this mapping. I find it very useful. Furthermore, to define a functional goal, (…) and orient yourself towards a common goal. Obviously, it's useful" [Physiotherapist 2].*

Physiotherapists found this particularly useful for building therapeutic relations. During the conversation, physiotherapists described validating the patient's responses and checking whether the information was correct: *" When we went through the questionnaire [patient profile], she said it corresponded well with what I had told her” [Patient 7].*

Although physiotherapists addressed approximately the same themes in an ordinary anamnesis as when using the patient profile, they usually did so in a less systematic and comprehensive order. As such, the CDSS helped to structure the dialogue and to focus on what is important during the consultation. Most physiotherapists thought the patients reflected well on their situation and said the patient profile made it easier to agree on where to focus. However, two physiotherapists underlined the need to be cautious, warning against becoming deterministic, especially if the patient presented with high and multifaceted symptoms: *“You can easily get stuck with prejudices. An expectation that this is not going to go well, that this is going to be difficult” [Physiotherapist 2].*

Patients described that they got a good first impression of the physiotherapists after using the tool, a sense of being seen, heard, and not treated as just another patient in line: *“You feel that someone has prepared and that you are seen in a different way when people are prepared when you arrive” [Patient 06].* One patient found the physiotherapist’s excitement for the CDSS motivating since he explained the CDSS in a language she understood and involved her by letting her watch the screen and participate in assessments. Others also appreciated how the CDSS helped align the patient-therapist mutual understanding of the current problem and how to proceed with the treatment: *“I liked best that we have a common picture of me and the plan ahead” [Patient 10].*

Visualising the patient profile on the screen was pointed out by patients as a facilitator if they struggled to explain the problems or express themselves. Patients with high symptom severity in the patient profile stated that they did not become scared or depressed since they were already aware of their situation. Instead, it led to constructive conversations with the physiotherapist, who was interested, responsive and empowered them with explanations and a better understanding of the situation.

#### 4) Patient involvement

Although the interviews showed that patient involvement in the different parts of the CDSS varied greatly, all patients felt they participated and could elaborate in discussions around their patient profiles. The patients also appreciated to participate in deciding on the activity for the patient-specific functional scale (PSFS) [41] and the main goal for the treatment together with the physiotherapist. The physiotherapists highlighted the dialogue on defining the activity and determining goals as useful. By involving patients in defining a treatment goal and an important patient-specific function, the physiotherapists believed the patients increased their awareness of the visit and the purpose of the treatment.

The patient involvement varied for the patient matching. Some patients described actively participating in the patient selection process, discussing treatment suggestions, and deciding if something should be omitted or added to the suggested treatment. One patient allowed to take an active part in the process stated that she felt more included and more on the level of the physiotherapist when deciding on treatment: “*You feel more included and on the same level. Even though she is a professional, I feel that she is understanding and speaks in a language that I understand, I feel that she is warm and that she cares, and as you also get to watch the screen and participate, it makes for a good experience” [Patient 10].* Some pointed out the opportunity to question the treatment suggested by the physiotherapist: *“It can be nice to ask a few questions and get a good explanation of why you are getting the treatment you are getting” [Patient 7].* However, one patient found it difficult to participate and relate to a system she had not seen before, and several others stated that they relied on the physiotherapist´s expertise.

Even though patients felt included in decisions, some wished they had spent more time on the part of the CDSS suggesting treatment. In contrast, one patient had no or little expectation of participating in the process of choosing treatment and was surprised when she had to be an active part herself. A few patients had not seen the treatment decision screen at all. Thus, some were unaware of this part of the CDSS, whereas others said the physiotherapist prepared the matching beforehand. As such, some patients were merely presented with the result without seeing similar patients, with no discussions or joint decisions on the patient’s preferences: *“She had found a match that was ready. She had peeked into several, and found the one that matched best” [Patient 9]. *This was, in some cases, reflected in the therapist interviews:* “The patient was involved and was the main architect in defining the main goal of treatment. And I think they do it with a little support from me, while I am more responsible for how we will get to that goal. So there, I'm probably not good enough to involve the patient” [Physiotherapist 3].*

While all physiotherapists told of exploring the matching and treatment suggestions beforehand without the patients, how and if they chose to involve the patients differed for various reasons. Some differences seems related to the individual therapists who told of how they generally involved patients, this spanned from revising and discussing all information together, showing the matching while having decided the treatment beforehand, to not showing the matching at all. However, the therapists also underlined the need to adjust their approach to the patient at hand, and when revising patient interviews, it became clear that patients visiting the same therapist experienced their involvement in the matching differently. For instance, one therapist told of barely involving patients in the matching, and not at all in treatment decisions from the CDSS. Whereas one patient treated by this therapist described being asked what feels right for her, another believed the treatment part of the CDSS was intended only for the therapist and said no discussions regarding choice of treatment had occured: *"It's certainly a support for the therapist. I am more uncertain whether the patient should be involved" [Patient 11].*

Some physiotherapists admitted they had potential for improvement regarding patient involvement, while others said that patients generally agreed on the selected treatment. The physiotherapists stated that the CDSS gave support for their own choice of treatment rather than functioning as a tool for making shared decisions with the patient: *"You need to use it like any other tool you have in the clinic, as part of the treatment tools you have, either to support you or to require some reflection on the treatment methods" [Physiotherapist 4].*

While some physiotherapists used the CDSS to support treatment already decided, others described it as a tool for focusing more on treatment within a biopsychosocial framework. Most physiotherapists described the treatment recommendations as supporting or substantiating their current practice while helping to reassess their thinking of best practice: *“The treatment given was quite similar to what I had already started, so it’s a decision support for me” [Physiotherapist 3].* However, one physiotherapist believed it could spread uncertainty if the treatment description differed from their own view. While most physiotherapists expressed openness about learning and applying new treatments as suggested by the CDSS, some also communicated uncertainties in addressing certain issues (e.g., mental health and sleep). On the other hand, one physiotherapist was more sceptical and, to some degree, unwilling to adapt to other suggestions.

#### 5) Recovery expectations

Several patients found the CDSS promising in identifying suitable treatment based on other similar patients. One patient described how the CDSS provided her with reassurance: *" (…) the reassurance that someone else had been through it and that the treatment has worked" [Patient 9].* Having one’s problems contrasted against someone else, maybe experiencing worse problems, also felt reassuring for some. However, this was not the case if the CDSS could not find similar optimal patients: *“It was a 20–30 years difference between me and the best match. So, then I wondered if I had the health of a 60-year-old” [Patient 9].*

Several patients found the three months patients’ trajectory graphs very motivating. Knowing their situation would improve over time was reassuring: *“I looked at some graphs that one person hadn't improved in the first few weeks, but then he had a great improvement after a while. I remember I noticed that” [Patient 12].* Also, the trajectories were seen as helpful in visualising how one variable, such as pain, could be constant, whereas others such as coping or functioning could improve over the same time. One physiotherapist pointed out that the trajectories might give patients unrealistic expectations, questioning their usefulness: *“Can it create unrealistic expectations when they look at the good progress, and after three months, we are sitting there, and they are much worse off?” [Physiotherapist 2].* However, the other physiotherapists believed the patient trajectories could be used to create positive beliefs and recovery expectations.

## Discussion

This study summarise the development and explore the acceptance and use of a CDSS for managing common MSK pain disorders. The results showed that physiotherapists and patients found the CDSS to be a preparatory and exploratory tool, facilitating the therapeutic relationship and promoting a biopsychosocial approach. Patient involvement varied for the different parts of the CDSS, especially in the treatment decision phase. Physiotherapists described using the CDSS predominantly to support their treatment choices rather than learning from it and adapting treatment based on similar past patients with successful outcomes.

Both patients and physiotherapists found the first part of the CDSS acceptable, describing it as easy to understand, exciting, informative, well-structured, and compatible with ordinary practice. Although user acceptance is critical for CDSSs, it has been identified as a shortcoming in implementing CDSSs in clinical care [23]. Physiotherapists in our study experienced good consultation workflow through the entire consultation with the patient and an acceptable amount of time in front of a computer screen. Other studies have found barriers caused by workflow interference, increased computer handling time, time and effort to enter patient data, and the experience of being interrupted by many and often incorrect reminders [42–46]. The absence of such disadvantages in the present CDSS might result from the lack of active reminders or alerts, the fact that clinicians were physiotherapists with traditionally more time for each consultation, and that the CDSS information was predominantly collected from the patients before the consultation. Thus, physiotherapists use just a minimum of time and effort to enter patient data. In addition, filling in questionnaires beforehand was experienced as a preparatory assignment which facilitated patients’ awareness, making them reflect on how factors like sleep, work and social participation influenced their health and increased awareness of what topics to focus on with the physiotherapist. This is in accordance with other studies on patient self-awareness, where questions about health issues make patients aware of their challenges and the severity of their problems [47, 48].

The patient profile was perceived as particularly useful for building the therapeutic relationship, and getting a comprehensive view of the patient as a whole was described as a good start for communication. The patients had a sense of being seen and heard and not treated just like another patient in line. This is consistent with person-centred care [5], where each patient is recognised as a unique person in a biopsychosocial framework and receives an individualised approach where communication is central to achieving a therapeutic relationship [49, 50]. In contrast to our study, others have experienced that CDSSs distract from the therapeutic relationship [44, 51]. The CDSSs in these studies provided alerts and reminders or were diagnostic tools, in contrast to the CDSS in our study, where the patient and the physiotherapist used it together for shared decision-making.

Involving the patient in decisions is fundamental to person-centred care and is characterised as a process where health personnel and patients work together to agree on healthcare choices [52]. Although shared decision-making is strongly embedded in health care, its effect is not reported for patient-reported outcomes in MSK pain [53]. We found much patient involvement in parts where patients could elaborate on self-reported information and be specific towards goal settings based on their preferences and context. However, the patient involvement and acceptance of the treatment decision phase were divided. Some physiotherapists found patient involvement problematic in this phase due to confusing or irrelevant information from the CDSS needing thorough explanations for patients to understand, unsuitable treatment recommendations or that recommendations were too sparse or extensive to learn from. Such barriers are in accordance with earlier work reporting that CDSSs provide too many or irrelevant recommendations [46]. The varying experience of acceptance and the less-than-optimal involvement of patients in comparing their profile to previous patients and deciding on treatment may in part reflect the physiotherapist's perception of their ability or desire to be involved. The physiotherapists themselves also explained filtering what was displayed depending on whether they believed the information would benefit the patient. The opportunity to omit parts of the CDSS unfortunately also allowed the physiotherapists to circumvent shared decision-making with the patient. This may explain why patients experienced different degrees of involvement in the treatment decision phase of the CDSS, from being unaware of it, not spending enough time on it, to actively discussing the suggested treatment. However, the variation in patient involvement also seems to differ according to physiotherapists to some extent. While two physiotherapists told of having involved patients throughout the CDSS, one showed matching and treatment to some patients while having decided the treatment beforehand, and the remaining physiotherapist told of barely involving patients in the matching at all. Due to the small sample size, it is however not possible to make any inferences as to why the physiotherapists differ in their practice, e.g. according to their characteristics. Patients experiencing more involvement reported feeling more included, and some highlighting the opportunity to question the treatment. However, several patients also stated having no or little expectation of participating in the process of choosing treatment or simply relying on the physiotherapist´s expertise. Patient’s reliance on the physiotherapist has been reported previously [54], and studies have also described that patients exhibit a dependence on the physiotherapist, preferring that they make the decisions [55, 56]. The various aspects influencing how the system is used (or not) should be studied further. For instance, in-depth attention to the patient-physiotherapist interaction may provide valuable insight into why the system is deemed inappropriate for some patients and how shared decision-making is performed.

It has been recommended to focus more on prognostic factors to inform treatment decisions and improve treatment outcomes in MSK pain [6]. Recovery expectations and positive beliefs are such factors [57–59]. In the present study, both physiotherapists and patients believed that recovery expectations and reassurance could increase if patients compared themselves with symptom severity and treatment trajectories of similar patients. However, trajectories for similar patients were used in only some consultations.

### Strengths and limitations

We included sixteen participants altogether, twelve patients and four physiotherapists. Although the physiotherapists were heterogeneously represented with different sex, ages, clinical experience, and physiotherapy specialities, the low number of physiotherapists is a limitation to the study that may have affected the content validity. That said, much can be learned from a small number of participants if open-ended questions giving more and richer data are used in the interview process [60]. In this case, interviews with physiotherapists lasted approximately 1.5 h, allowing them to provide extensive information. Three researchers (FG, AB, NEK) with different expertise conducted the interviews. FG and AB are physiotherapists with experience developing the CDSS. NEK (PhD) is a social anthropologist with experience conducting qualitative interviews. FG and AB were responsible for the physiotherapist’s education, which could have influenced and biased the responses of the physiotherapists. However, comprehensive interview guides were followed, and at least two interviewers, always including the experienced interviewer, were present. A strength, considering the credibility of the study, is that the results have been discussed within several research groups, with researchers from different professions giving feedback on the interpretation of the results. Other aspects to ensure trustworthiness for credibility, dependability and confirmability is that we present the codes and themes (Table 2), that some of the researchers were experienced physiotherapists working with MSK pain disorders in the clinic and that researchers independently coded the first interviews before comparing and reaching agreement during the analysis phase. A limitation of the study is that several patients only had experience with some components of the treatment decision screen whereas others had none, providing limited knowledge of patients' experiences and making it challenging to compare acceptance and use on this part of the CDSS. However, that few patients had seen the full system was also an important finding, revealing why some physiotherapists did not involve their patients in deciding treatment due to finding treatment suggestions unsuitable for the patient’s problem. This may reflect an overall limitation of the smaller than optimal number of previous patients in the CDSS (*n* = 105) or that the treatment decision screen contains excessive information functionalities. In a final CDSS the case base will expand as the self-reported data from the new patient, description of the treatment and patient outcome will be retained for future problem-solving.

### Implications

The perceived usefulness and ease of use of the CDSS among physiotherapists and patients provide important information for future CDSS development. In contrast to others [44, 51], the patient profile of the CDSS was found to be very useful for the therapeutic relationship, and the comprehensive patient mapping was described as a good start for communication. Essential for this positive user acceptance was that patients provided extensive information about their health issues in advance of the consultation and thus saving time and that participants were prepared having answered questionnaires (patients) or looked at the patient profile before the first consultation (physiotherapists). Whereas others have reported impaired clinical workflow and increased computer handling time using CDSSs [42–46], this was solved by collecting almost all data before the first consultation in the present study. In addition, the patient and physiotherapist discussed the patient's symptom severity from the patient profile together, using it as a normal anamnesis facilitated by having potentially difficult topics visualised.

Using the CBR methodology, new patients are matched to previous, similar patients and displayed in the CDSS. When new patients can compare themselves with similar successful patients, physiotherapists and patients believe recovery expectations and reassurance increase. However, this part of the CDSS might have a potential that we did not fully exploit and should be further studied. Although patients were involved in discussing their symptom severity and finding treatment goals, they were less involved in treatment decisions. Extensive education of physiotherapists for securing treatment fidelity should not be underestimated to build understanding and trust in the CDSS. Future studies should explore how patients could be more involved in deciding treatments.

## Conclusions

This study summarise the development and explore the acceptance and use of a CDSS for managing common MSK pain disorders in primary care physiotherapy. We identified themes for ease of use and user acceptance. Important findings were that the CDSS was valued as a preparatory and exploratory tool with a comprehensive assessment of the patients’ biopsychosocial profile, facilitating the therapeutic relationship. As for deciding treatment, the CDSS was mainly used by physiotherapists to support their treatment choices rather than involving patients to a greater extent in deciding treatment and learning from previous successful patients. The results from this study will be used to adapt the CDSS further. A randomised controlled trial is planned to evaluate the effectiveness of the CDSS in primary care physiotherapy.

### Supplementary Information

**Additional file 1. **Interview guides.

## Data Availability

The data used in the current study are available from the corresponding author on reasonable request.

## Abbreviations

MSK: Musculoskeletal
CDSS: Clinical decision support system
AI: Artificial intelligence
CBR: Case-based reasoning
CORE-Q: Consolidated criteria for reporting qualitative research
MSK-HQ: The Musculoskeletal Health Questionnaire
ÖMSPQ: The Short Form Örebro Musculoskeletal Pain Screening Questionnaire
PSFS: The Patient-Specific Functional Scale
