# Supplementary material for: Acceptance and use of a clinical decision support system in musculoskeletal pain disorders – the SupportPrim project
Source: BMC Med Inform Decis Mak. 2023 Dec 19;23:293. doi: 10.1186/s12911-023-02399-7 (PMC10731802; doi:10.1186/s12911-023-02399-7)
Supplement: Supplementary file 1 — Additional file 1. Interview guides. [file 12911_2023_2399_MOESM1_ESM.docx]

**Interview guide therapist**

**Scheme**

- Do you use any questionnaires to screen patients as part of your regular practice?

**Baseline information**

- Do you look at the patient´s baseline information before the first consultation?
- How do you experience having this information before meeting the patient?
- What is your perception of the patient profile? (e.g., amount and relevancy of information, time needed to go through it with the patient, comprehensibility, explainability, alignment with patient's/your perception of their problem)
- How do you use it? (e.g., with/without the patient, if the information deviates from the patient's/your perception of the patient’s problem)

**Baseline examination**

- How do you use the baseline examination? (e.g., number of tests, use of time, consultation flow)
- How do you find goals and patient-specific activity? (e.g., agreement/different perspectives)

**Patient match**

- What is your perception of the patient matching? (e.g., accuracy of match, amount and relevancy of information, time needed to go through it with the patient, comprehensibility, need for explanation, alignment with own/patient perceptions)
- How do you use it? (with/without the patient, level of interaction, agreement/disagreement, which information is relevant for choosing treatment, who chooses treatment, use of time)
- How did you use the trajectories? How did you use the treatment suggestions? (e.g., together, alone, before, after)
- How should the description of the treatment be? (e.g., which information, for the patient/ for the clinician, level of abstraction and detail, terminology, prospect of transferability)

**In general**

- How was the education? Do you feel that you know how to use the CDSS? Are you able to explain the CDSS to patients?
- How many consultations did you use to go through all parts of the CDSS? Could you have done everything in one consultation?
- How were the consultations compared to usual practice? (e.g., time, reassurance for doing the right things)
- Does the CDSS facilitate patient involvement and shared decision-making? Why/why not?
- Do you change the way you use the CDSS for different patients? How?
- Does the use of the CDSS change your role as a clinician? How can the CDSS contribute to your treatment?
- Could the CDSS be more useful for some patients/therapists?
- Should something be changed? (e.g., facilitators, barriers, flexible enough)
- Would you recommend this to colleagues?
- Is there anything I haven't asked you that you think is important or thought I would ask?

**Interview guide patient**

**Questionnaire before the first consultation**

- How much time did you use to fill out the questionnaire? (e.g., reasonable length, too many questions, could there have been more)
- Did you understand the questions? (e.g., difficult/easy to understand or/answer, relevant to you and your health, something missing)
- Did you think differently about your health situation after filling out the questionnaire?
- Had your therapist looked at your answers before the first consultation? How did you feel about that?

**Patient profile**

- Was the information easy or hard to understand? Did you get a proper explanation?
- How was the use of colours?
- Did the information correlate with how you feel? Did anything surprise you?
- How was the amount of information? (e.g., a lot, little, adequate)
- Did you use sufficient time on this part?
- How did you explore the CDSS? (e.g., watching the screen together, sitting next to each other)
- Do you have any thoughts on what it was like to see this representation of your health situation afterwards?

**Baseline examination**

- Did the therapist do the examination and fill in the information underway? How did this affect the consultation flow?
- How did you find goals and the patient-specific activities?

**Patient match**

- How did you experience seeing your profile in the radar plot (e.g., easy to understand, sufficient explanation, match with your experience of how you feel)
- How did you select a match and treatment? (e.g., similar characteristics, graphs describing the patient’s course, treatment recommendations, motivation for the type of treatment, easy/difficult, therapists´ or your choice/joint discussion, agree/disagree)
- Did you spend enough time on this part?
- Did you feel that you understood the information?

**In general**

- How many times have you been to your physiotherapist?
- How far did you get in the first consultation? Could the whole CDSS be done in one consultation?
- Did you feel that the therapist had the skills needed to use the CDSS and provide answers to your questions?
- How did the consultation(s) differ from your prior physiotherapy experiences (if any)?
- How did you feel communication between you and your therapist was affected by using the CDSS?
- What did you like the most? What could be changed?
- Do you think this type of consultation is better suited for some than others?
- Is there anything I haven't asked you that you think is important or thought I would ask you?
